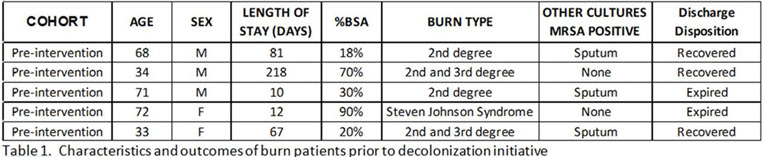# Decrease in MRSA Bacteremia After Implementation of Intranasal Mupirocin Decolonization Protocol

**DOI:** 10.1017/ash.2021.113

**Published:** 2021-07-29

**Authors:** Angela Beatriz Cruz, Jennifer LeRose, Teena Chopra, Mara Cranis, Lori Cullen, Kenisha Evans, Monica Meyer, Lavina Jabbo, Judy Moshos, Rudolph Valentini

## Abstract

**Background:** Methicillin-resistant *Staphylococcus aureus* (MRSA) remains a key pathogen in burn patients and is associated with increased morbidity and mortality. Disruption of skin barrier exposes these individuals to a myriad of infections. Various decolonization approaches, including chlorhexidine baths and intranasal mupirocin, have shown favorable outcomes in preventing MRSA infections in this cohort. **Methods:** In August 2020, a mupirocin decolonization protocol was implemented in Michigan’s largest trauma-level 1 burn intensive care unit. All patients admitted to the burn unit received daily intranasal mupirocin for the initial 5 days of hospitalization. We compared MRSA bacteremia rates per 1,000 patient days from January–July 2020 to those after August 2020. A hospital-acquired MRSA bacteremia infection was defined as a positive blood culture after hospital day 3. Patient characteristics and hospital course were collected through medical chart review. A 2-tailed *t* test was used for analysis. **Results:** We identified 5 cases of hospital-onset MRSA bacteremia and no cases of community-onset MRSA bacteremia. On average, there were 2.6 cases per 1,000 patient days before mupirocin implementation and 1.0 cases per 1,000 patient days after mupirocin implementation (*P* = .26) (Figure 1). In this patient cohort, the average total body surface area burned was 45.6% (range, 18%–90%), and 60% (n = 3) of patients had sputum culture positive for MRSA prior to developing bacteremia (Table 1). Also, 2 patients (40%) with MRSA bacteremia died. Notably, the patient in the postintervention cohort was admitted in July, prior to implementation. **Conclusions:** Implementation of a decolonization protocol with intranasal mupirocin in burn-surgery patients markedly decreased the incidence of MRSA bacteremia in this cohort. This is the first study to evaluate the use of mupirocin as a decolonizing agent in burn victims. Continued long-term surveillance is recommended, and this strategy has potential for application to other high-risk cohorts.

**Funding:** No

**Disclosures:** None

**Figure 1.**
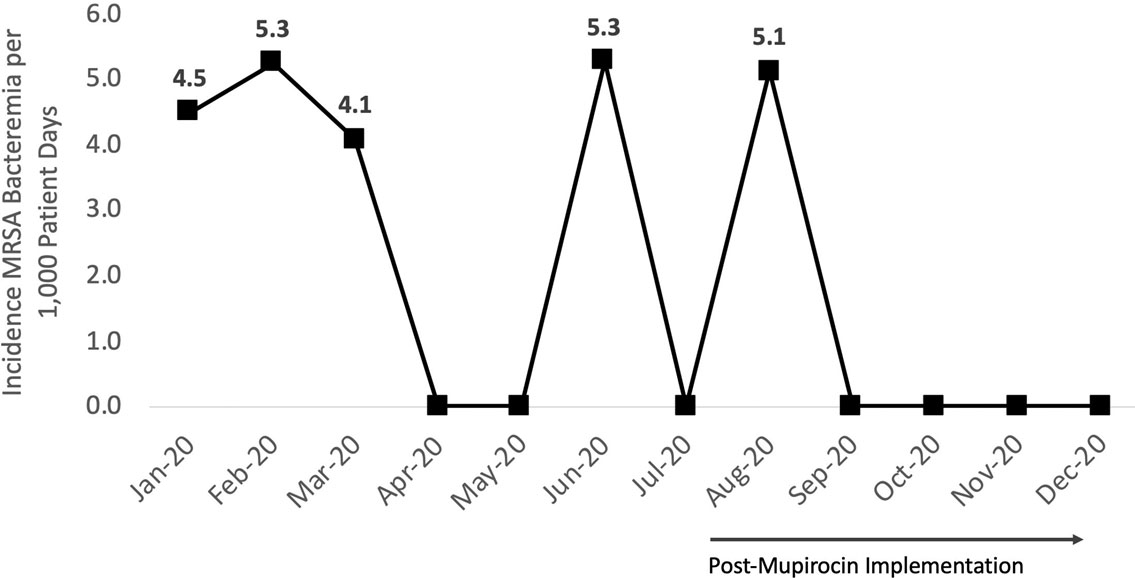

**Table 1.**